# Beyond Independence: Cultural Values and Dependence Avoidance Among Older Adults in Japan and South Korea

**DOI:** 10.1093/geroni/igaf122.3250

**Published:** 2025-12-31

**Authors:** Tomoko Ikeuchi, Tzu-Yu Lin, Donghee Han

**Affiliations:** Takachiho University, Tokyo, Tokyo, Japan; Kaohsiung Medical University, Kaohsiung, Kaohsiung, Taiwan (Republic of China); Research Institute of Science for the Better Living of the Elderly, Busan, Busan, Republic of Korea

## Abstract

Japan and South Korea are experiencing rapid depopulation and population aging, with a growing number of older adults living alone while striving to remain independent. In these East Asian cultures, maintaining harmony and avoiding burdening others are highly valued. This study examines whether the desire to avoid dependence differs from a preference for independence. Data were collected in February 2025 through online surveys of 800 Japanese and 600 Korean participants (Mage=58.0, SD = 10.2, range=40-81; 50.9% women). Multiple regression analyses revealed that, in both countries, women and older age were associated with greater reluctance to depend on or inconvenience people outside their in-group. However, when considering in-group members (i.e., family, close friends, colleagues), this association was found only for older adults in Japan. Similarly, a preference for independence was linked solely to older age in Japan. These findings suggest that women and older individuals may refrain from seeking support from those outside their in-group, such as community members or government service providers, even when their in-group members are no longer dependable. Furthermore, a reluctance to depend on others may not necessarily equate to a preference for independence. Given that women in these nations have some of the world’s longest life expectancies, our findings highlight the need for accessible support systems tailored to older women while respecting their cultural values. This study underscores the importance of culturally sensitive approaches to aging support in East Asian societies, recognizing the distinction between avoiding dependence and preferring independence.

